# Could Sterile *Aedes albopictus* Male Releases Interfere with *Aedes aegypti* Population in Reunion Island?

**DOI:** 10.3390/insects13020146

**Published:** 2022-01-29

**Authors:** Harilanto Felana Andrianjakarivony, David Damiens, Lucie Marquereau, Benjamin Gaudillat, Nausicaa Habchi-Hanriot, Louis-Clément Gouagna

**Affiliations:** 1 Microbes, Evolution, Phylogeny and Infection (MEΦI), IHU—Méditerranée Infection, Aix-Marseille University, 13007 Marseille, France; afhfelana@gmail.com; 2 Microbiologie Environnementale Biotechnologie (MEB), Mediterranean Institute of Oceanography (MIO), 13009 Marseille, France; 3 Institut de Recherche pour le Développement (IRD), UMR MIVEGEC (CNRS/IRD/Université de Montpellier), Maladies Infectieuses et Vecteurs, Ecologie, Génétique, Evolution et Contrôle, BP 64501, CEDEX 5, 34394 Montpellier, France; lucie.marquereau@ird.fr (L.M.); b.gaudillat@cyroi.fr (B.G.); nausicaa.habchi-hanriot@ars.sante.fr (N.H.-H.); louis-clement.gouagna@ird.fr (L.-C.G.); 4 IRD Réunion/GIP CYROI (Recherche Santé Bio-Innovation), 97490 Sainte Clotilde, Reunion Island, France

**Keywords:** sterile insect technique, rhodamine, heterospecific mating

## Abstract

**Simple Summary:**

The Sterile Insect Technique consisting of inundative and repeated releases of sterile males that induce sterility in the wild population is currently tested as a new strategy to control *Ae. albopictus.* Considering that *Ae. albopictus* and *Ae. aegypti* thrive in sympatry in some regions, and that mating between both species is known to occur at low rates, we hypothesize that releasing millions of sterile male *Ae. albopictus* in could affect female *Ae. aegypti* reproduction. To study this potential mating interference, the newly established marking technique has been applied using the rhodamine B that is administered to males through sugar meal. It is internally incorporated into the mosquito’s male body and during mating, the rhodamine is transferred into the females. In laboratory-based experiments rhodamine marking proved to be a powerful means of detecting mating in females of both *Aedes* species, whatever the mating crosses between males and females. Some mated females were able to lay eggs, but all were not viable. However, despite the promiscuity of the adults in small experimental cages, 95% of the female *Ae aegypti* showed no evidence of insemination following mating with sterile male *Ae. albopictus*, suggesting that in the field, an inundative and repeated releases of sterile male *Ae. albopictus* will not influence the reproduction of female *Ae aegypti*.

**Abstract:**

In Reunion Island, the feasibility of an *Aedes albopictus* control program using the Sterile Insect Technique (SIT) is studied. Because, in some regions, *Ae. albopictus* is living in sympatry with *Aedes aegypti*, the impact of releasing millions of sterile male *Ae. albopictus* on female *Ae. aegypti* reproduction needs to be assessed. Thus, to study the potential heterospecific matings, a marking technique using rhodamine B has been used. Rhodamine is given in solution to male mosquitoes to be incorporated into the male body and seminal fluid and transferred during mating into the bursa inseminalis and spermathecae of females. The presence of rhodamine in females occurred in 15% of cases when *Ae. aegypti* females were offered non-irradiated *Ae. albopictus* males, 5% when offered irradiated *Ae. albopictus* males and 18% of cases in the inverse heterospecific matings. Moreover, our results also showed that these matings gave few eggs but were not viable. Finally, the results showed that whatever the type of mating crosses, females in cages previously crossed with males of another species can re-mate with males of their species and produce an equivalent amount of egg compared to females only mated with conspecific males. Despite the promiscuity of the males and females in small cages for three days, heterospecific mating between sterile male *Ae. albopictus* and female *Ae aegypti*, 95% of the females have not been inseminated suggesting that in the field the frequency satyrization would be very low.

## 1. Introduction

In Reunion Island, *Aedes aegypti* and *Aedes albopictus* species are living in sympatry in a few limited locations [1]. *Aedes albopictus* is present abundantly on the coastal areas of the island while *Ae. aegypti* is present in very isolated and limited places [1,2]. *Aedes aegypti* is a pantropical species that spread centuries ago on islands in the Southwestern Indian Ocean (SWIO) including Reunion Island. Based on phylogenetic analyses of the mtDNA-CO1 sequences, Delatte et al. [3] hypothesized that at least two waves of invasion of *Ae. albopictus* occurred on the islands in SWIO. The first one probably occurred dozens of centuries ago alongside the human colonization of Madagascar [4]. The second wave of invasion occurred in the 1990s [3]. This most recent invasion by *Ae. albopictus* in Reunion Island has probably induced the decline of the *Ae. aegypti* population as observed in southeastern USA and Bermuda [5,6,7]. In Reunion Island, *Ae. albopictus* is the main vector of dengue 2017–2019 epidemy [8,9]) and2006 chikungunya outbreak [10]. A program of *Ae. albopictus* control using the Sterile Insect Technique (SIT) as a part of an Area-Wide Integrated Pest Management (AWIPM) has been thus started. The SIT is based on an inundative and repeated releases of sterile males that induce sterility in the wild population, and consequently suppress the target pest species [11].

In the location where both *Aedes* species are present, the impact of the release of millions of sterile males *Ae. albopictus* on female *Ae. aegypti* reproduction is unknown. In nature, heterospecific matings are known to occur at low rates; between 3.6% for male *Ae. albopictus* mating female *Ae. aegypti* and 1.4% for male *Ae. aegypti* mating female *Ae. albopictus* in Florida for example [12], and on average 2.17% for *Ae. aegypti* and 1.22% for *Ae. albopictus* observed by Bargielowski et al. [13]. If a high number of sterile male *Ae. albopictus* are released in such region, an increase of heterospecific mating rate could be hypothesized with a potential impact on the *Ae. aegypti* population dynamics.

In heterospecific matings, satyrization is defined as any kind of interspecific interaction during the process of mate acquisition that adversely affects the fitness of at least one of the species with or without sperm transfer, with or without fertilization, with or without progeny, readily occurred [14,15]. In heterospecific mating between male *Ae. albopictus* and female *Ae. aegypti*, two major situations that could influence female *Ae. aegypti* reproductive behavior have been observed: the transfer of sperm during the mating without production of offspring [16] or transfer of semen (male accessory gland proteins) only with no sperm [12,17]. Usually, these situations could induce in female *Ae. aegypti* a refractory period to further mating with conspecific while the reverse case is not true [12]. In addition, male *Ae. albopictus* aggressive and non-selective behavior could lead to a female *Ae. aegypti* harassment [18]. This could impair sugar and blood-feeding behavior and impeach optimum reproduction thereof [18]. Such satyrization between two species has already been proposed as a method for the biological control of pests and vectors [14].

Several experiments have been performed about satyrization between *Ae. albopictus* and *Ae. aegypti* with variable results [12,17,19,20]. Results are inconsistent, probably due to the fact that every paper has been performed with different strains of *Ae. albopictus* and *Ae. aegypti* and with strains kept in labs for different numbers of generations. Moreover, the duration and intensity of contact between both species in the field could also probably modify the degree of satyrization. These observations suggest carrying a satyrization rate estimation before any sterile males mass release program in situations where the two species coexist.

To observe the potential heterospecific mating that could occur between sterile male *Ae. albopictus* and female *Ae. aegypti* on Reunion Island, a marking technique using the rhodamine B newly developed on *Ae. aegypti* was used [21]. Considered as a suitable alternative to the use of fluorescent powder as a marker, rhodamine B is a thiol-reactive fluorescent dye, that has proven useful for the internal marking of insects. Typically, male mosquitoes acquired the mark after feeding on a sugar diet supplemented with a 0.4% rhodamine solution. It incorporates internally into the male body and seminal fluid and can be transferred during mating into the bursa inseminalis (BI) and spermathecae of females. Sterile and non-sterile *Ae. albopictus* males previously marked with rhodamine were used in different cross-mating treatments with conspecific and heterospecific females to estimate the intensity of mating interference that could occur in the field following the release of sterile of *Ae. albopictus* males where *Ae. aegypti* populations exist.

## 2. Materials and Methods

### 2.1. Mosquito Preparation

The *Ae. albopictus* strain used in this study was originated from a colony originally established from eggs collected in the field at Saint Marie (Reunion Island) in 2014 and was maintained at the insectarium for several generations before these experiments were performed in 2018. The *Ae. aegypti* strain was originated from field collections in Trois Bassin (Reunion Island) and was colonized in the laboratory for four generations (F4) before the experiment.

For both species, batches of one thousand five hundred eggs from each population were counted into rearing trays (30 × 40 × 10 cm). Upon hatching, larvae were reared at a density of ~0.5 larvae/mL, in a controlled climatic chamber (Versatile Environmental Test Chamber MLR-350H, Sanyo electric Co., Ltd., Osaka Japan) at 29 °C and a photoperiod of 12:12 (L:D). They were fed with 5, 7, 7 and 5 mL per tray of a solution at 7.5% (wt:vol) slurry of diet (50% ground rabbit-food and 50% ground fish-food Tetramin, Tetra, Herrenteich Germany) on days 1,2,3 and 4, respectively. From the fifth day to the seventh day, pupae that appeared were collected and segregated by sex to produce virgin males and females. After sex separation, male pupae were allowed to emerge into laboratory cages (30 × 30 × 30 cm) with constant access to a 5% sucrose solution [*w*/*v*]. Female pupae were first isolated in tubes (5 per tube) to check the accuracy of the sexing at the emergence and then transferred into laboratory cages.

### 2.2. Irradiation

To produce irradiated males, male pupae more than 30-h-old were irradiated at 35 Gy during 5 min with X-ray irradiator (Blood X-RAD 13-19, Cegelec, Paris, France) at the Blood bank coordinated by l’Etablissement Français du Sang (EFS) located at the Bellepierre hospital, Saint Denis. The irradiation dose applied was previously determined to effectively induce >99% sterility in the adult males irradiated at the pupal stage [22]. The irradiated male pupae were brought back to the laboratory, and left to emerge in Bugdorm cages (30 × 30 × 30 cm; MegaView, Taichung, Taiwan).

### 2.3. Rhodamine Marking

After emergence, males were maintained with the marking solution for four days. The marking solution was prepared in a 100 mL pot, by adding 320 mg of rhodamine powder to 80 mL of 5% sucrose solution (giving a 0.4% rhodamine solution) [21]. A funnel made with a filter paper was plunged in the marking solution allowing adults to take the sugar and the opening of the funnel was plugged by a piece of cork to prevent mosquitoes from drowning in the solution.

### 2.4. Mating Experiments

#### 2.4.1. Transfer of Materials during Conspecific and Heterospecific Mating

All the experimental males and females came from the same cohort that emerged on the same day. All mating experiments described below took place as followed: random batches of 25 four-day-old marked virgin males and 25 four-day-old virgin females were placed in the same Bugdorm cage (30 × 30 × 30 cm). They were allowed to mate for three days, under a climate-controlled insectary at 27 ± 2 °C, RH: 75 ± 2%, and 12L:12D regimen.

Six mating treatments were performed: three conspecific matings (virgin males and virgin females of the same species); non irradiated male × female *Ae. albopictus*, irradiated male × female *Ae. albopictus* and male × female *Ae. aegypti*, and three heterospecific matings (virgin males of one species and virgin females of the other species); non irradiated male *Ae. albopictus* × female *Ae. aegypti*, irradiated male *Ae. albopictus* × female *Ae. aegypti* and male *Ae. aegypti* × female *Ae. albopictus*. For each treatment, the experiment was repeated three times.

At the end of the reproductive period of three days, females were removed from the cage, dissected and their bursa inseminalis and their spermathecae examined under a microscope (Leica MZ6, X40) for the presence of sperm in order to determine whether there was insemination in cross-mating treatments.

Females were dissected in a saline solution (PBS [phosphate buffered saline], 137 mM NaCl, 2.7 mM KCl, 10 mM Sodium Phosphate dibasic, 2 mM Potassium Phosphate monobasic, pH = 7.4) on a slide under stereomicroscope. Bursa inseminalis and the three spermathecae were then transferred in a drop of 20 μL of a PBS containing DAPI (4′,6-diamidino-2-phenylindole dihydrochloride, 2 μg/mL) placed on another slide. DAPI was used to stain sperm and determine their presence in the bursa inseminalis or in the spermatheca. A cover slip was then placed upon the drop and observed under X-200 magnification using a fluorescence microscope (Olympus BX41, Center Valley, PA, USA). The double staining allowed the determination of the transfer of different material from the males: protein coming from the annex gland (with rhodamine B) and sperm coming from testes (with DAPI).

#### 2.4.2. Offspring Production after Conspecific and Heterospecific Mating

All the experimental males and the females came from the same cohort that emerged on the same day. All mating experiments took place in Bugdorm cages (30 × 30 × 30 cm) as followed: 50 four-day-old virgin males and 50 four-day-old virgin females were put in the same laboratory cage with continuous access to a 10% (*w*/*v*) sucrose solution for three days. Following a three-day mating period, females were blood-fed for two days. They were allowed to feed on 20 mL of cow blood through a collagen membrane (Hemotek company, Blackburn, UK) on the Hemotek^®^ system (PS6 Power Unit, Discovery Workshops, Accrington, Lancashire, UK) for 30 min. The use of cow blood provided by the slaughterhouse for mosquito feeding did not require any particular ethical clearance. Two days after blood feeding, plastic cups containing 100 mL of water and lined with strips of crepe filter paper were provided in each cage for oviposition paper for two days. The positive egg papers were then dried and stored for eight days in controlled laboratory conditions to allow egg maturation and the synchronization of hatching [23]. Eggs laid on individual ovistrips were then counted (using an optical microscope, Leica MZ6, x20) and then placed in one small container containing water in which dehydrated rabbit food (hay pellet, Compagnie des Grains du Capricorne, Le Port, Reunion Island) were added for hatching. The container was sealed for 24 h. Larvae L1 were then removed from each container, counted, and then placed into rearing trays and allowed to develop in the same conditions as that of the regular rearing regime (see above). The hatching rate was defined as the number of L1 larvae divided by the number of eggs counted. At the third stage, larvae were identified to determine the fathering and estimate offspring issued from heterospecific mating.

Eight mating treatments were performed: three conspecific mating treatments (virgin males and virgin females of the same species); non irradiated male × female *Ae. albopictus*, irradiated male × female *Ae. albopictus* and male × female *Ae. aegypti*, three heterospecific mating treatment (virgin males of one species and virgin females of the other species); non irradiated male *Ae. albopictus* × female *Ae. aegypti*, irradiated male *Ae. albopictus* × female *Ae. aegypti* and male *Ae. aegypti* × female *Ae. albopictus* and two negative controls with virgin females of both species alone (held under the same conditions in absence of males). For each treatment, the experiment was repeated three times from different batches.

#### 2.4.3. Influence of Heterospecific Mating on Subsequent Female Reproductive Behavior

After the egg-laying period, the same females from the three heterospecific mating treatments in the previous experiment (i.e., non irradiated male *Ae. albopictus* × female *Ae. aegypti*, irradiated male *Ae. albopictus*/female *Ae. aegypti* and male *Ae. aegypti* × female *Ae. albopictus*), were put in a cage with a group of 50 newly emerged (two-days-old) conspecific males. After three days, a time deemed necessary for mating, the females were again blood-fed for two days. Subsequently, the same protocol as described above was realized to determine egg production and hatch rate.

### 2.5. Statistical Analysis

The analysis of variance (ANOVA) with Tukey’s honestly significant difference post hoc tests and Student’s *t*-test were used to test differences in the average percentage of females marked with rhodamine and with sperm, mean number of eggs laid by females and egg hatching rate between experimental groups and among replicates. Prior to statistical analyses, data recorded as percentages were first arcsine-square root transformed to increase their fit to the normal distribution. All statistical analyses were carried out using Graphpad prism 7.

## 3. Results

### 3.1. Transfer of Materials during Conspecific and Heterospecific Mating

**Figure 1:**
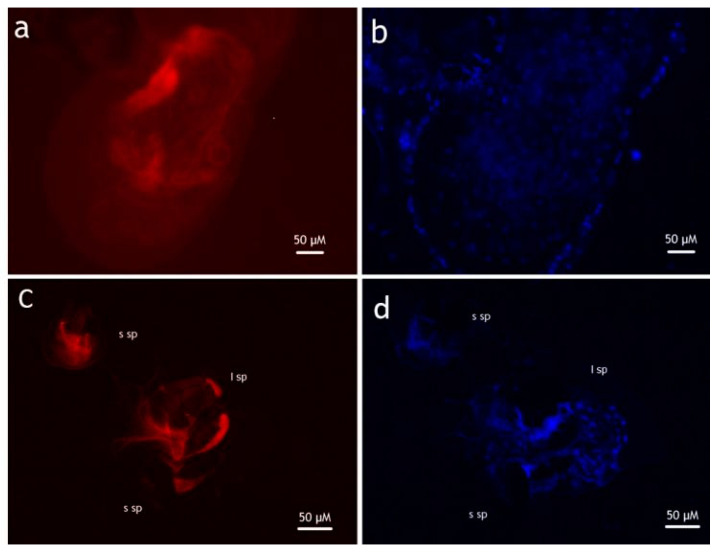
*Aedes albopictus* reproductive system observed after mating with virgin rhodamine-fed male (magnification 400×). (**a**). Bursa inseminalis marked with rhodamine (**b**). Same bursa inseminalis marked with DAPI (sperm are visible) (**c**). spermathecae marked with rhodamine (**d**). same spermathecae with DAPI. s sp indicate small spermatheca and l sp large spermatheca.

Table 1 presents the percentage of females marked with rhodamine and with sperm cells in spermathecae after seven-day contact with virgin males in the six mating treatments. The percentages of females marked with rhodamine were significantly different depending on the treatments (ANOVA test, F5,13 = 445.8, *p* < 0.001). For all conspecific crosses with fertile males, sperm were observed for each mating and all females were marked with rhodamine in the bursa inseminalis and in spermatheca (Figure 1a,b). However, mating with irradiated *Ae. albopictus* yielded a lower proportion of marked females with rhodamine. The difference was statistically significant compared to crosses with non-irradiated males. The number of marked females in conspecific mating was significantly different from heterospecific mating, regardless of species. In heterospecific mating, although there was no transfer of sperm whatever the treatments, some females were marked with rhodamine suggesting a transfer of protein from the accessory gland.

### 3.2. Offspring Production after Mating

The mean number of eggs and the hatching rate between the different treatments were compared in Table 2. The mean number of eggs produced by females in the different treatments was significantly different (ANOVA test, F7,15 = 13.3, *p* < 0.001). In cages with only virgin females, no female *Ae. albopictus* laid eggs while female *Ae. aegypti* were able to lay eggs in one cage but with no hatching. The means of the number of eggs in conspecific crosses were significantly different from heterospecific-mating regardless of species. In addition, there was no effect (*p* > 0.01) of irradiation on the mean number of eggs produced by females mated with the males in conspecific crosses and heterospecific mating. Hatch rates were significantly different depending on treatment (ANOVA test, F7,15 = 1412.5, *p* < 0.001), hatch rates of conspecific mating were high while were zero for heterospecific matings. Moreover, as expected, hatch rate of females *Ae. albopictus* mated with sterile males *Ae. albopictus* was very closed to zero.

### 3.3. Influence of Heterospecific Mating on the Later Female Reproductive Behavior

The results in Table 3 showed that there was no significant difference between the average number of eggs laid by females after conspecific mating alone and the numbers obtained after heterospecific mating and then mating with conspecific males in *Ae. albopictus* (*t* test, t = 0.0137, df = 3, *p* = 0.99). There was also no significant difference between the mean number of eggs of females *Ae. aegypti* mated with only male *Ae. aegypti* and mean number of eggs of females mated with non-irradiated or irradiated male *Ae. albopictus* first and then with *Ae. aegypti* (ANOVA test, F2,6 = 0.92849, *p* = 0.45). Moreover, whatever the treatment, there was no significant difference in hatch rate (ANOVA test, F4,10 = 2.259709, *p* = 0.13).

## 4. Discussion

In the current study, matings have been observed between *Ae. albopictus* and *Ae. aegypti*, since the presence of rhodamine in the bursa inseminalis proved the transfer of seminal fluid in 15% of cases when female *Ae. aegypti* were offered non-irradiated male *Ae. albopictus*, and 18% of successful mating had taken place when female *Ae. albopictus* were placed in cages in the presence of male *Ae. aegypti*. This transfer is less important for irradiated male *Ae. albopictus*, suggesting an effect of irradiation on the male’s ability to transfer its semen to a heterospecific female.

However, in heterospecific mating, no rhodamine markings were found in the spermatheca nor was sperm visible with DAPI coloration in both *Ae. albopictus* and *Ae. aegypti* females, as observed by Carrasquilla and Lounibos [17] and Maiga et al. [24]. The presence of semen in bursa inseminalis but not in spermatheca supports the suggestion of Vick [25] that the transfer of male accessory gland material could occur independently of sperm transfer in spermatheca. However, these results are different from Nasci et al. [19] who found that most female *Ae. aegypti* mated by males *Ae. albopictus* had some dead sperm in their spermathecae or Leahy and Craig [16] who observed 20% of females with sperm in spermatheca after three weeks of contact. Marcela et al. [20] found 8% of female *Ae. aegypti* with sperm in spermatheca after contact with male *Ae. albopictus* (in a cage with 20 males *Ae. albopictus* and 20 females *Ae. aegypti* for five days). One hypothesis is that we could have missed the few numbers of sperm stored in spermatheca which could be supported by the presence of egg-laying. However, egg-laying could be due to the autogenous production of eggs without sperm by *Ae. aegypti* as observed in the control cages where virgin females were blood-fed.

In heterospecific mating between male *Ae. aegypti* and female *Ae. albopictus*, the same observations have been performed with no spermatozoa found to be DAPI-marked in spermatheca but with a production of very few eggs. Female *Ae. albopictus* are known to have low levels of autogeny (see review in [26]) which could explain the presence of eggs in heterospecific cages and the absence in control ones. Maiga et al. [24] also observed a low number of eggs after the same heterospecific matings with no presence of sperm observed in the spermatheca. However, this egg-laying could be from a female that has stored a very small amount of sperm that was not observed in the DAPI coloring experiment. Such a presence of sperm in the spermatheca has been observed in previous studies [27] as observed by Marcela et al. [22] after contact between 20 male *Ae. aegypti* and 20 female *Ae. albopictus* for five days. Fourteen percent of these females were found with spermatozoa in spermatheca and were able to produce few eggs but none of these eggs gave offspring. In heterospecific mating between male *Ae. albopictus* and female *Ae. aegypti*, females were able to lay eggs but with no offspring. As *Ae. albopictus*, *Ae. aegypti* females have autogenous activity [28] as observed in one cage of controls. Our results agree with those of Harper and Paulson [29] and Maiga et al. [24] who demonstrated that the mating of *Ae. aegypti* and *Ae. albopictus* gave eggs but they are not viable. Most studies state that no offspring are produced from these crosses [30].

There is no reason to exclude the possibility of heterospecific mating with the transfer of accessory gland (MAG) products (as peptides and other elements), thereby regulating the mating interaction between the species that we experimentally considered here. The magnitude of the implication of such a mechanism could explain the results observed in our study. In *Ae. albopictus* [31,32,33], but also in *Ae. aegypti* [34,35], MAG products are known to be sufficient to induce oviposition and refractoriness to remating. For example, *Ae. albopictus* MAG makes female *Ae. aegypti* refractory to mating with conspecific males by decreasing their diurnal locomotor activity [36]. Moreover, among the MAG, Head Peptide-1 (HP-1), a short neuropeptide hormone discovered in males of *Ae. aegypti* that prevents females from mating with another male, is also present in male *Ae. albopictus* [37]. HP-1 isolated from males of *Ae. albopictus* and injected in females of *Ae. aegypti* reduced re-mating [37]. Carrasquilla and Lounibos [17] showed that *Ae. aegypti* females previously mated with *Ae. albopictus* males were refractory to a second mating with the males of their species, even without sperm in their spermatheca. All these observations suggest a possible mechanism of satyrization of *Ae. albopictus* males on females of *Ae. aegypti*. However, under our conditions and for a small population, females previously exposed with males of another species and then presented to congeners produced a similar number of eggs with a similar hatch rate, leading to the same number of offspring as females mated with only congeners. This is in contrast with a previous report of reduced numbers of viable eggs in *Ae. Aegypti* females exposed to *Ae. albopictus* males and later mated with their conspecific males [17]. Although such a reduced egg hatch rate might presumably reflect the cost of satyrisation, no such changes were observed in our study. Consistent with a recent study reported by Maiga et al. [24], this suggests that in our conditions, even if interspecies induction of refractory period occurred, it is very limited and has no impact on the final product of mating: the quantity of offspring. Despite this, in the context of SIT that involve the inundative release of sterile males to selectively control the target species, future research under natural conditions should aim at gaining a quantitative understanding of the potential impact of an increased heterospecific mating rate following sterile male release on the dynamics of coexisting *Aedes* species and subspecies (if any). Elucidating the role of heterospecific mating is a first step in dissecting the overall contribution of sterile male release to reproductive control of non-target sympatric and closely related species.

## 5. Conclusions

In our experiments, even if there were heterospecific mating between *Ae. albopictus* males and *Ae. aegypti* females, 96% of the females have not been inseminated despite the promiscuity of the males and females in small cages for three days. Identified structural incompatibilities in the genitals, as well as responses to flight sounds, could result in barriers to the coupling of these two species [16]. The heterospecific mating probably occurs by mistake due to the failure of species recognition [15]. Knowing that rates of heterospecific mating in laboratory conditions are expected to be higher compared to in the field since in cages females are harassed due to crowding and have limited escaping options, we can hypothesize that the probability of heterospecific mating in the field in Reunion Island is very low. Moreover, the probability of heterospecific encounter events that took place around the host [38] will vary with host availability. The probability will be low if human hosts are rare or animal hosts frequent due to the zoophily of *Ae. aegypti* and the anthropophily of *Ae. albopictus* [39]. These hypotheses were confirmed by Tripet et al. [12] who reported only a 1.8% of satyrization phenomenon in *Ae. aegypti* in Florida. In addition to the expected low probability of mating between the two species, no effect of mating between male *Ae. albopictus* and female *Ae. aegypti* have been observed on egg production in our results suggesting a very limited satyrization probability. No control of the *Aedes aegypti* population would be possible with an SIT program controlling *Ae. albopictus*. To avoid a resurgence of large *Ae. aegypti* population in case of *Ae. albopictus* suppression, an SIT program on *Ae. aegypti* should be performed before or concurrently with the *Ae. albopictus* program, only in the regions where both species are living in sympatry.

## Figures and Tables

**Table 1:** Mean percentage (±Standard Deviation) of females marked with rhodamine and with sperm (colored by DAPI) after seven-days contact between males and females in the six treatments. The columns with the letters represent the results of the post Hoc Tukey test after ANOVA test on mean percent of marked females, different letters indicated a significant difference. Values with the same letter were not significantly different.

Rhodamine Marked Males	Female	Mean Percent of Marked Females	Post Hoc Test Tukey for Marked Females	Mean Percent of Females with Sperm
*Ae. albopictus*	*Ae. albopictus*	100 ± 0.0	a	100 ± 0.0
*Ae. albopictus sterile*	*Ae. albopictus*	91.1 ± 3.1	b	100 ± 0.0
*Ae. aegypti*	*Ae. aegypti*	100 ± 0.0	a	100 ± 0.0
*Ae. albopictus*	*Ae. aegypti*	14.9 ± 6.5	c	0 ± 0.0
*Ae. albopictus sterile*	*Ae. aegypti*	4.2 ± 4.2	c	0 ± 0.0
*Ae. aegypti*	*Ae. albopictus*	17.9 ± 10.4	c	0 ± 0.0

**Table 2:** Mean number (±Standard Deviation) and mean hatch rate (±Standard Deviation) of eggs laid by females mated during the eight treatments. The columns with the letters represent the results of the post Hoc Tukey test, different letters indicated a significant difference. Values with the same letter were not significantly different.

Rhodamine Marked Males	Female	Mean Number of Eggs	Post Hoc Test Tukey for Number of Eggs	Mean Hatch Rate	Post Hoc Test Tukey for Hatch Rate
-	*Ae. albopictus*	0.0 ± 0.0	b	0.0 ± 0.0	c
-	*Ae. aegypti*	20.3 ± 35.2	b	0.0 ± 0.0	c
*Ae. albopictus*	*Ae. albopictus*	302.0 ± 164.0	a	84.7 ± 1.9	a
*Ae. albopictus sterile*	*Ae. albopictus*	254.7 ± 48.2	a	0.1 ± 0.2	c
*Ae. aegypti*	*Ae. aegypti*	183.0 ± 83.5	a	70.0 ± 3.9	b
*Ae. albopictus*	*Ae. aegypti*	5.33 ± 5.5	b	0.0 ± 0.0	c
*Ae. albopictus sterile*	*Ae. aegypti*	0.0 ± 0.0	b	0.0 ± 0.0	c
*Ae. aegypti*	*Ae. albopictus*	11.0 ± 11.0	b	0.0 ± 0.0	c

**Table 3:** Mean number (±Standard Deviation) and mean hatch rate (±Standard Deviation) of eggs laid by females mated first with rhodamine marked heterospecific males and then with conspecific males.

Rhodamine Marked Males	Females	Males	Mean Number of Eggs	Hatch Rate
First mating		Second mating		
*Ae. albopictus*	*Ae. albopictus*		302.0 ± 164.0	84.7 ± 2.0
*Ae. aegypti*	*Ae. albopictus*	*Ae. albopictus*	303.3 ± 60.8	86.1 ± 8.2
*Ae. aegypti*	*Ae. aegypti*		183.0 ± 83.5	70.3 ± 3.9
*Ae. albopictus*	*Ae. aegypti*	*Ae. aegypti*	130.3 ± 34.0	76.5 ± 3.3
*sterile Ae. albopictus*	*Ae. aegypti*	*Ae. aegypti*	130.0 ± 29.9	70.3 ± 9.8

## Data Availability

All relevant data are contained within the article—Table 1, Table 2 and Table 3.
